# Human Transcriptomic Meta-Analysis Identifies Immune-Redox Remodeling and Impaired Vascular Wall Homeostasis in Abdominal Aortic Aneurysm

**DOI:** 10.3390/ijms27167257

**Published:** 2026-08-14

**Authors:** Morgan Engel, Sarah Voskamp, Mary McIntosh, Robert E. Akins, Heather P. Park, Jennifer S. Nelson

**Affiliations:** 1 Department of Surgery, University of Central Florida College of Medicine, Orlando, FL 32827, USA; mo922522@ucf.edu (M.E.); sarah.voskamp@nemours.org (S.V.); ma044709@ucf.edu (M.M.); 2 Department of Cardiovascular Services, Nemours Children’s Hospital, Orlando, FL 32827, USA; 3 Department of Biomedical Research, Nemours Children’s Hospital, Wilmington, DE 19803, USA; robert.akins@nemours.org; 4 Tanner Vascular Surgery, Tanner Medical Group, Tanner Health System, Carrollton, GA 30117, USA; hpark@tanner.org

**Keywords:** abdominal aortic aneurysm, transcriptomics, gene expression, inflammation, oxidative stress, Ingenuity Pathway Analysis, STARGEO

## Abstract

Abdominal aortic aneurysm (AAA) is a progressive vascular disease in which no pharmacological therapy has consistently reduced expansion or rupture risk, highlighting the need to better define reproducible molecular programs in human aneurysmal tissue. Publicly available human abdominal aortic tissue microarray datasets were identified using the Search, Tag, Analyze Resource for Gene Expression Omnibus (STARGEO) platform. Three independent datasets comprising 324 total samples, including 278 aneurysmal abdominal aortic tissue samples and 46 non-aneurysmal control samples, were included. Transcriptomic meta-analysis identified 2331 differentially expressed genes in AAA tissue relative to non-aneurysmal controls. Upregulated genes were enriched for immune and inflammatory signaling, oxidative stress regulation, and extracellular matrix remodeling. Downregulated genes included regulators of vascular smooth muscle cell structure, cell–matrix adhesion, cytoskeletal organization, and metabolic homeostasis. Human AAA tissue demonstrates a reproducible inflammatory–oxidative transcriptional program characterized by leukocyte recruitment, cytokine amplification, innate immune sensing, redox remodeling, protease-mediated extracellular matrix degradation, and suppression of vascular smooth muscle cell and matrix maintenance programs. These findings support a model of AAA as an immune–redox remodeling disease in which inflammatory, oxidative, proteolytic, structural, and failed resolution pathways converge to destabilize the aortic wall.

## 1. Introduction

Abdominal aortic aneurysm (AAA) is a permanent, focal dilation of the abdominal aorta, commonly defined as an aortic diameter ≥ 1.5 times its expected diameter or an absolute diameter ≥ 30 mm. The global prevalence of AAA is roughly 0.9%, with marked male predominance and strong associations with age and smoking [1]. Early-stage disease is typically asymptomatic, with most AAA detected incidentally through imaging or screening programs. The clinical significance derives from the very high risk of rupture once the aneurysm exceeds the repair threshold: for aneurysms ≥ 55 mm, five-year rupture risk can reach 25–40%, with observed mortality rates exceeding 80% in affected patients [2,3]. Accordingly, current management emphasizes serial imaging surveillance until an aneurysm reaches a size at which intervention is indicated. This surveillance interval represents a biologically active period in which disease progression could, in principle, be modified. However, despite extensive investigation, no pharmacological therapy to date has consistently slowed aneurysm expansion or reduced rupture risk in clinical trials [4].

AAA pathogenesis reflects a convergence of extracellular matrix (ECM) failure, maladaptive vascular remodeling, and sustained immune activation. Aneurysmal tissue shows progressive degradation of elastin and collagen, imbalance between proteolytic enzymes and their inhibitors with heightened matrix metalloproteinase (MMP) activity, and apoptosis or phenotypic switching of vascular smooth muscle cells (VSMCs) that normally maintain wall integrity [5]. These structural changes occur alongside chronic inflammation involving dense infiltration of innate and adaptive immune cells, with macrophage- and T-cell-rich adventitia and media, and frequent intraluminal thrombus that can serve as a reservoir for inflammatory mediators [5]. In parallel, increasing evidence links AAA to dysregulated cellular metabolism. Tissue and circulating metabolic profiles have suggested enhanced glycolysis and dysregulated lipid metabolism in AAA, consistent with a disease state that links immune activation to metabolic reprogramming and intersects with broader cardiometabolic risk processes [5]. This molecular overlap aligns with the shared epidemiology between AAA and other cardiovascular disorders, including associations with hypertension, dyslipidemia, and atherosclerotic disease [5].

Genetic evidence supports AAA as a strongly inheritable disorder. Family and twin studies estimate the rate of heredity approaches 70%, and large genome-wide association meta-analyses have identified approximately 140 independent susceptibility loci. Many of those loci implicate pathways related to lipid metabolism, vascular wall structure, and inflammatory signaling, but collectively they explain only a fraction of overall risk. Thus, additional genetic contributors, context-dependent regulatory effects, and other gene environment interactions remain undefined [6,7,8]. This gap is clinically important because it likely reflects characterized molecular drivers that could be leveraged for risk stratification by marker development or therapeutic targeting.

In the context of persistent therapeutic limitations and incomplete mechanistic resolution, integrative transcriptomic approaches provide an opportunity to identify reproducible molecular mechanisms in human aneurysmal tissue. In this study, we performed a cross-platform meta-analysis of publicly available human abdominal aortic gene expression datasets obtained from the National Center for Biotechnology Information’s (NCBI) Gene Expression Omnibus (GEO). Using the Search, Tag, Analyze Resource for GEO (STARGEO) platform, we integrated three independent microarray cohorts comparing aneurysmal and non-aneurysmal abdominal aortic tissue to define differentially expressed genes consistently associated with AAA. We then applied pathway enrichment, upstream regulator, and network-based analyses using Ingenuity Pathway Analysis (IPA) to characterize dominant biological processes and candidate regulatory drivers underlying the aneurysmal transcriptomic signature. By prioritizing concordant gene-level and pathway-level alterations across independent datasets, this systems-level approach aimed to refine mechanistic hypotheses and nominate molecular targets for further translational investigation.

## 2. Results

### 2.1. Differential Gene Expression in AAA Tissue

**Figure 1:**
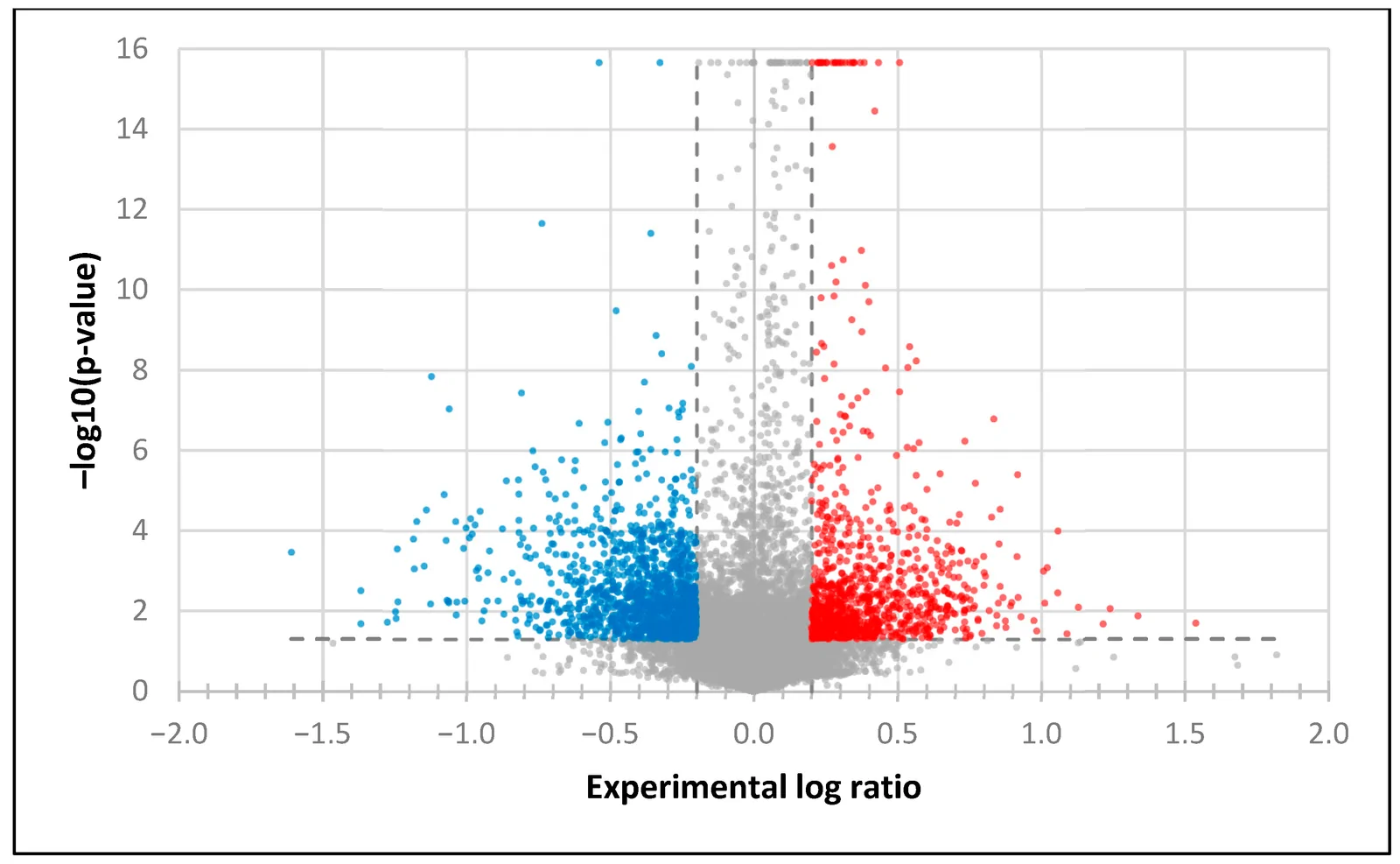
Volcano plot of differential gene expression identified in AAA tissue relative to non-aneurysmal control tissue. Each point represents one of the 22,112 genes evaluated in the STARGEO meta-analysis. The *x*-axis shows the experimental log ratio, and the *y*-axis shows −log_10_ of the raw meta-analysis *p*-value. Red points indicate upregulated genes meeting both *p* < 0.05 and an experimental log ratio > 0.2, whereas blue points indicate downregulated genes meeting both *p* < 0.05 and an experimental log ratio < −0.2. Gray points did not meet both primary differential expression criteria. Vertical dashed lines indicate experimental log ratios of −0.2 and 0.2, and the horizontal dashed line indicates *p* = 0.05.

Transcriptomic meta-analysis comparing AAA tissue with non-aneurysmal controls integrated expression results for 22,112 genes across three independent human datasets generated from distinct microarray platforms. Of these, 2331 genes met inclusion criteria of *p  *<  0.05 and absolute experimental log ratio > 0.2 (Table S1), comparable to the extent of differential expression reported in prior AAA transcriptomic analyses [9]. Experimental log ratios ranged from −1.609 to +1.538, indicating substantial bidirectional remodeling within the aneurysmal aortic wall. The 10 most upregulated and downregulated genes ranked by experimental log ratio are listed in Table 1 and a volcano plot of all significant differentially expressed genes identified in this transcriptomic meta-analysis is seen in Figure 1.

As a sensitivity analysis, Benjamini–Hochberg correction was applied across all 22,112 gene-level meta-analysis *p*-values. Of the 2331 genes meeting the primary differential expression criteria, 938 remained significant at aa false discovery rate (FDR)-adjusted *p*-value ≤ 0.05 and an absolute experimental log ratio > 0.2. Of the 10 most upregulated genes, three (*UCP2*, *LTB*, and *MMP12*) remained significant after FDR correction, whereas five of the 10 most downregulated genes (*PPP1R3C*, *SPEG*, *ITGA8*, *FHL5*, and *KCNA5*) remained significant. FDR-adjusted *p*-values for these genes are included in Table 1. The complete list of all differentially expressed genes and their corresponding FDR-adjusted *p*-values is provided in Table S4.

The most upregulated genes were enriched for immune and inflammatory signaling pathways (*FOSB*, *IL1B*, *FCN1*, *CD83*, *CCR7*, *LTB*) [10,11,12,13,14], oxidative stress and reactive oxygen species (ROS) regulation (*NCF1*, *UCP2*) [15,16], and ECM remodeling (*MMP12*, *MMP25*) [17,18], whereas several downregulated genes were associated with vascular smooth muscle structural integrity and metabolic regulation (*SPEG*, *ITGA8*, *KCNA5*, *SLC25A4*) [19,20,21,22]. Among the most highly expressed genes, *FOSB* (experimental log ratio 1.538), a component of the AP-1 transcription factor complex, has been linked to immune cell infiltration and VSMC phenotypic modulation in aneurysmal disease [23]. *IL1B* (experimental log ratio 1.239), a central pro-inflammatory cytokine, promotes MMP production and VSMC apoptosis, contributing to aneurysmal wall degeneration [24,25]. Similarly, *UCP2* (experimental log ratio 1.058), a mitochondrial regulator of oxidative stress, has been reported to be enriched in macrophage populations within human AAA tissue and associated with aneurysm progression in experimental models [26]. Consistent with these findings, *NCF1*, a component of the nicotinamide adenine dinucleotide phosphate (NADPH) oxidase complex, has been implicated in ROS-mediated signaling pathways that influence vascular inflammation and aneurysm formation [27].

Genes associated with ECM remodeling also were prominent among the upregulated transcripts. *MMP12* (experimental log ratio 1.020), a macrophage-produced elastolytic protease, has previously been localized to regions of medial elastin fragmentation in AAA specimens [28]. *MMP25* (experimental log ratio 1.089) has likewise been reported to regulate macrophage migration, NF-κB activation, and chemokine signaling in experimental models of vascular inflammation and aneurysm development [29]. In parallel, immune cell trafficking signals were represented by *CCR7* (experimental log ratio 1.012), a chemokine receptor associated with lymphocyte recruitment and immune cell accumulation in aneurysmal tissue [30,31]. Consistent with this inflammatory signature, multiple chemokine genes, including *CXCL8*, *CCL3*, *CCL4*, and *CXCL1*, also were upregulated. *CXCL8* (experimental log ratio 1.008), for example, has been detected at elevated levels in aneurysmal tissue and is implicated in neutrophil recruitment and inflammatory amplification within the aneurysmal wall [32,33]. Recent transcriptomic analyses have further identified neutrophil-associated genes, including *LTB*, as key mediators of immune cell recruitment and neutrophil extracellular trap (NET)-related tissue injury in AAA [34]. RUNX3 (runt-related transcription factor 3; experimental log ratio 0.974), a transcription factor implicated in vascular remodeling, was also upregulated and has been reported to modulate VSMC phenotypic transitions in AAA [35]. Collectively, these findings reinforce the enrichment of gene networks involved in immune activation, leukocyte recruitment, transcriptional regulation, and ECM degradation.

In contrast, the most strongly downregulated genes included markers of vascular smooth muscle structural integrity, cytoskeletal organization, and metabolic regulation. Regulator of G-protein signaling 5 (*RGS5*; experimental log ratio −0.875), a marker of quiescent VSMC, was markedly reduced, consistent with prior reports describing depletion of *RGS5*-high smooth muscle cell populations in aneurysmal tissue [31]. Insulin-like growth factor binding protein 2 (*IGFBP2*; experimental log ratio −0.960) was also decreased, in agreement with studies demonstrating reduced expression in AAA tissue [32]. Fibulin-5 (*FBLN5*; experimental log ratio −1.006), a key mediator of elastic fiber assembly, showed diminished expression, consistent with impaired ECM integrity in aneurysmal degeneration [33]. Tissue inhibitor of metallopeptidase inhibitor 4 (*TIMP4*; experimental log ratio −0.980), an endogenous inhibitor of MMP, was similarly reduced, reflecting the protease–antiprotease imbalance characteristic of aneurysmal pathology [34]. Additional downregulated genes included vascular smooth muscle structural regulators (*SPEG*, *ITGA8*, *KCNA5*) [36,37,38,39], metabolic enzymes (PPP1R3C, SLC25A4) [40,41], and transcriptional regulators such as ZBTB16 [28], collectively indicating loss of vascular smooth muscle stability and metabolic homeostasis in aneurysmal tissue.

### 2.2. Top Canonical Pathways

To contextualize differentially expressed genes within coordinated biological programs, canonical pathway enrichment analysis was performed using IPA. Enrichment significance was assessed using −log_10_*p*-value and predicted activation states were inferred using IPA activation z-scores based on the directionality of constituent genes.

Canonical pathway analysis of the 2331 AAA-associated genes demonstrated marked enrichment of immune and inflammatory signaling pathways in aneurysmal tissue relative to controls. Neutrophil degranulation emerged as the most significantly enriched pathway (−log_10_*p*-value 27; ratio 0.254; z-score 6.636), reflecting broad upregulation of genes involved in innate immune effector responses [34]. Additional innate immune pathways included leukocyte extravasation signaling (−log_10_*p*-value 25.5; ratio 0.361; z-score 3.20), phagosome formation (−log_10_*p*-value 21.1; ratio 0.201; z-score 3.06), and Fcγ receptor-mediated phagocytosis (−log_10_*p*-value 14.6; ratio 0.387; z-score 2.20), indicating coordinated activation of adhesion, migration, and phagocytic machinery. Pathogen-associated cytokine signaling also demonstrated strong predicted activation (−log_10_*p*-value 20.5; ratio 0.247; z-score 5.047). Similarly, pattern recognition receptor (PRR) signaling (−log_10_*p*-value 12.2; ratio 0.286; z-score 4.082), *TREM1* signaling (−log_10_*p*-value 11.2; ratio 0.367; z-score 3.674), and interferon-γ (*IFNG*) signaling (−log_10_*p*-value 12.2; ratio 0.347; z-score 3.773) were significantly enriched and predicted to be activated, consistent with amplification of innate immune sensing and inflammatory signaling genes.

Adaptive immune signaling pathways also were enriched. Both Th1 (−log_10_*p*-value 20.7; ratio 0.400; z-score 5.032) and Th2 (−log_10_*p*-value 21.3; ratio 0.383; z-score 4.323) pathways were predicted to be activated, indicating concurrent engagement of T helper cell-mediated immune responses. These patterns are consistent with transcriptional signatures of T-cell activation and antigen-driven immune responses in aneurysmal tissue [42]. 

**Figure 2:**
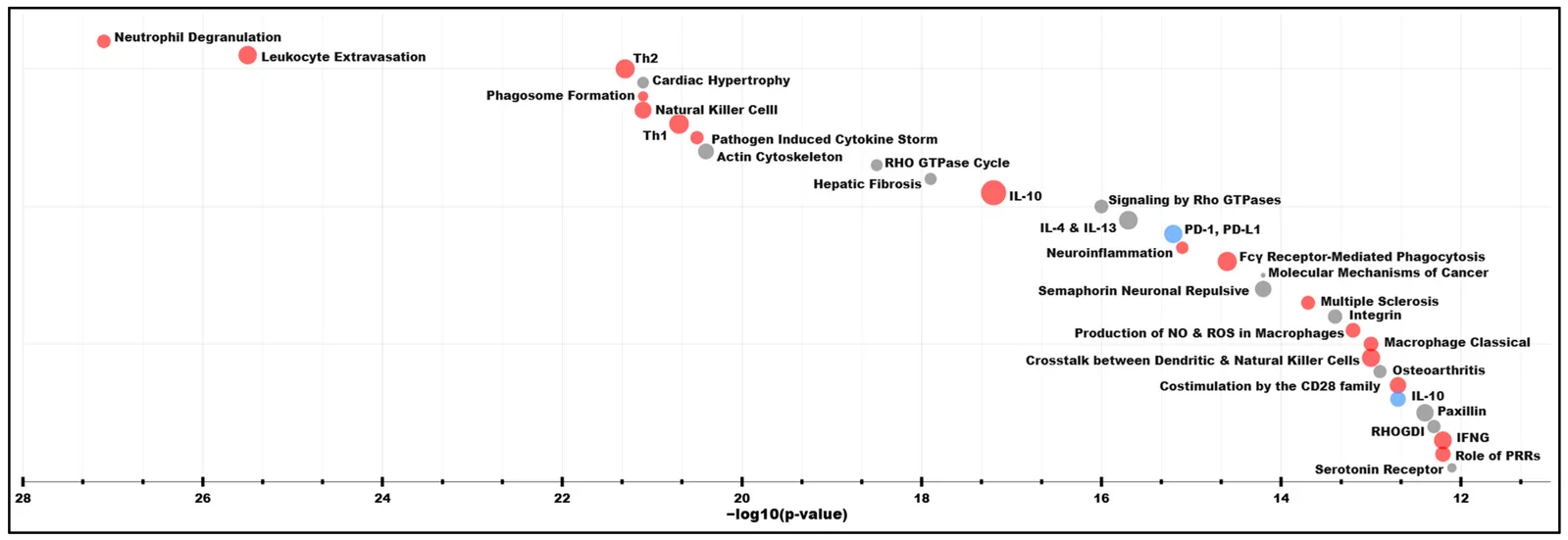
Bubble plot of the top 32 canonical pathways enriched in AAA tissue relative to non-aneurysmal controls, ranked by −log_10_(*p*-value). Top enriched canonical pathways in AAA tissue relative to non-aneurysmal controls identified by IPA. The bubble plot displays the 32 most significant pathways ranked by −log_10_*p*-value. Bubble size represents the proportion of pathway genes that are differentially expressed (ratio), and color indicates predicted activation state based on IPA z-scores (red, activated; blue, inhibited; gray, no prediction).

In contrast, several pathways related to ECM integrity and immunoregulatory control demonstrated negative activation states. ECM organization was significantly enriched with a negative z-score (−log_10_*p*-value 10.3; ratio 0.308; z-score −2.959), consistent with downregulation of structural matrix components. Anti-inflammatory regulatory pathways, including IL-10 signaling (−log_10_*p*-value 12.7; ratio 0.29; z-score −2.596) and PD-1/PD-L1 checkpoint signaling (−log_10_*p*-value 15.2; ratio 0.36; z-score −3.656), also showed predicted inhibition, suggesting impaired immunoregulatory control and resolution of inflammation within aneurysmal tissue. The top enriched canonical pathways are summarized in Figure 2, which illustrates the predominance of immune and inflammatory signaling pathways alongside suppression of immunoregulatory processes. The complete results are provided in Table S2.

### 2.3. Upstream Regulators and Regulator Effect Modeling

To identify potential regulatory drivers underlying the observed transcriptional changes, upstream regulator analysis was performed using IPA.

The top predicted activated upstream regulators included polyinosinic:polycytidylic acid (poly[I:C]; z-score 9.45), lipopolysaccharide (*LPS*; z-score 9.33), *IFNG* (z-score 8.22), tumor necrosis factor (*TNF*; z-score 7.97), and stimulator of interferon response cGAMP interactor 1 (*STING1*; z-score 7.51). These regulators are associated with innate immune activation and cytokine-mediated inflammatory signaling. Immunoglobulin signaling was also predicted to be activated (z-score 4.37), consistent with increased Fc receptor-associated gene expression within the dataset. 

In contrast, the most strongly predicted inhibited regulator was dexamethasone (z-score −8.71), along with transcriptional regulators associated with anti-inflammatory or immunoregulatory functions, including cbp/p300 interacting transactivator with Glu/Asp rich carboxy-terminal domain 2 (*CITED2*), immunity-related GTPase M (*IRGM*), interferon regulatory factor 2 binding protein 2 (*IRF2BP2*), and three prime repair exonuclease 1 (*TREX1*), all with z-score ≤ −5.3. Inhibition of dexamethasone reflects an expression profile opposing glucocorticoid-mediated transcriptional suppression, suggesting attenuation of anti-inflammatory signaling within aneurysmal tissue.

Overall, upstream regulator analysis indicates coordinated activation of innate immune and cytokine-driven signaling pathways alongside reduced activity of anti-inflammatory regulatory programs. These findings are consistent with the pathway enrichment results and support a model of sustained inflammatory signaling in AAA tissue. The complete results are provided in Table S3.

## 3. Discussion

### 3.1. Principal Findings and Integrated Disease Framework

This meta-analysis of 324 human aortic samples demonstrates that AAA tissue exhibits coordinated transcriptional reprogramming characterized by immune activation, redox remodeling, and loss of vascular structural integrity. Rather than identifying a single dominant gene or isolated pathway, these findings support a systems-level model in which aneurysmal tissue shifts away from vascular wall homeostasis toward sustained immune-mediated remodeling, oxidative injury, ECM degradation, and impaired structural repair.

Across differential gene expression, canonical pathway enrichment, and upstream regulator modeling, the aneurysmal transcriptome was characterized by activation of leukocyte recruitment, neutrophil and macrophage effector programs, cytokine signaling, PRR activity, and redox-associated pathways. In parallel, downregulated genes and inhibited pathways pointed toward loss of VSMC contractile programs, impaired ECM organization, and reduced immunoregulatory restraint. Together, these findings support a model in which AAA progression reflects both active inflammatory tissue injury and inadequate compensatory repair.

Because these data derive from bulk tissue, the observed expression patterns likely reflect both altered cellular composition and altered activation states within resident vascular cells and infiltrating immune populations. Therefore, upregulation of immune-associated transcripts may reflect leukocyte enrichment, increased activation of immune cells, inflammatory reprogramming of resident vascular cells, or a combination of these processes. This interpretation is consistent with prior single-cell and spatial transcriptomic studies demonstrating expansion of neutrophils and inflammatory monocyte/macrophage subsets in AAA tissue alongside VSMC depletion or phenotypic switching [43,44]. 

### 3.2. Immune Activation and Leukocyte Effector Programs

The dominant transcriptional signal identified in this meta-analysis was the activation of immune and leukocyte effector programs within aneurysmal tissue. Canonical pathway analysis demonstrated marked enrichment of neutrophil degranulation, leukocyte extravasation signaling, phagosome formation, Fc receptor-mediated phagocytosis, PRR signaling, *TREM1* signaling, IFNG signaling, and Th1/Th2 immune pathways. Together, these pathways indicate that the aneurysmal wall is not simply characterized by isolated inflammatory gene expression, but by coordinated activation of immune cell recruitment, innate immune sensing, phagocytic activity, cytokine amplification, and adaptive immune engagement.

The prominence of neutrophil degranulation was particularly notable, as it emerged as the most significantly enriched canonical pathway in AAA tissue. This finding supports growing evidence that neutrophils are active contributors to aneurysmal remodeling rather than passive bystanders [43,44]. Neutrophil-associated pathways may promote vascular injury through release of proteolytic enzymes, ROS, inflammatory cytokines, and NET-related mediators [45]. Although the present analysis does not directly measure NET formation, the enrichment of neutrophil degranulation and related inflammatory pathways suggests neutrophil effector mechanisms may contribute to the inflammatory and proteolytic environment of the aneurysmal wall.

Gene-level findings reinforced this immune-dominant pattern. Upregulation of *IL1B* supports activation of a central pro-inflammatory cytokine axis involved in leukocyte recruitment, inflammatory amplification, and matrix-degrading responses [46,47,48]. Increased expression of *MMP12* and *MMP25* further links immune activation to ECM remodeling, as these proteases are associated with macrophage- and leukocyte-mediated tissue degradation [49,50]. Similarly, upregulation of *NCF1* connects phagocyte activation to oxidative burst and ROS-mediated vascular injury [51,52,53]. These findings suggest that inflammatory signaling, proteolysis, and oxidative stress are transcriptionally coupled within AAA tissue.

Additional upregulated genes point toward broad activation of innate immune sensing and leukocyte trafficking. *FCN1*, a pattern recognition molecule involved in lectin complement pathway activation, supports engagement of innate immune recognition mechanisms [11,54,55]. *LTB* may contribute to inflammatory signaling and lymphoid organization [14,56], while chemokine-associated genes including *CCR7*, *CXCL8*, *CCL3*, *CCL4*, and *CXCL1* suggest active recruitment and organization of immune cells within the aneurysmal wall. In this context, the upregulation of *CCR7* and *CD83* is especially relevant because these genes suggest involvement of antigen-presenting cells and adaptive immune signaling, consistent with the observed enrichment of Th1 and Th2 pathways [57,58,59]. 

Collectively, these findings support a model in which AAA tissue contains an organized and sustained immune response involving both innate and adaptive components. Innate immune pathways appear to dominate the transcriptional signature through neutrophil degranulation, phagocytosis, PRR signaling, *TREM1* signaling, and complement-associated sensing. At the same time, *CCR7*/*CD83* expression and Th1/Th2 pathway activation suggest chronic adaptive immune engagement may help sustain inflammation over time. Together, these immune signatures provide the upstream inflammatory context for the redox remodeling and structural wall degeneration discussed in Section 3.3 and Section 3.4.

### 3.3. Oxidative Stress as an Amplifier of Inflammatory Remodeling

Oxidative stress emerged as a major component of the AAA transcriptional profile and appears to function as a mechanistic bridge between immune activation and structural degeneration of the aortic wall. Rather than representing a separate process, redox remodeling is closely integrated with the leukocyte effector programs described above. Infiltrating neutrophils and macrophages are major sources of ROS within inflamed vascular tissue [60,61], and the upregulation of redox-associated genes in this analysis suggests oxidative injury may amplify inflammatory signaling, protease activation, and VSMC dysfunction within the aneurysmal wall.

At the gene level, upregulation of *NCF1* provides one of the strongest links between immune activation and oxidative stress. *NCF1* encodes p47phox, a regulatory component of the NOX2 NADPH oxidase complex, which is activated during phagocyte respiratory burst [62,63]. Increased *NCF1* expression suggests enhanced phagocyte-derived ROS generation in AAA tissue [60,61]. This finding aligns with the enrichment of neutrophil degranulation, phagocytosis, and innate immune effector pathways, supporting a model in which recruited leukocytes contribute directly to redox-mediated vascular injury.

Upregulation of *UCP2* further supports mitochondrial involvement in the aneurysmal redox response. *UCP2* regulates mitochondrial oxidative stress and metabolic adaptation, particularly in inflammatory cell populations [64,65]. In the context of AAA, increased *UCP2* expression may reflect altered mitochondrial metabolism within infiltrating immune cells and stressed vascular cells. Although *UCP2* may serve compensatory or context-dependent roles, its upregulation supports the broader conclusion that mitochondrial redox regulation is altered within the aneurysmal microenvironment.

The predicted activation of *STING1* provides an additional connection between oxidative stress, cellular injury, and innate immune amplification. Mitochondrial dysfunction and oxidative DNA damage can promote release of mitochondrial or nuclear DNA fragments into the cytosol, where they may activate cytosolic nucleic acid-sensing pathways such as cGAS-STING. *STING1* activation can then promote interferon signaling, cytokine production, inflammatory cell recruitment, and MMP induction [66,67]. In this framework, oxidative stress may result from inflammation and reinforce innate immune signaling through damage-associated nucleic acid sensing.

Functionally, ROS can promote aneurysmal remodeling through several converging mechanisms. Redox-sensitive signaling enhances inflammatory cytokine production, activates matrix-degrading proteases, and contributes to VSMC phenotypic switching and apoptosis [60,62]. These effects directly connect oxidative stress to the major structural findings of this analysis, including upregulation of matrix remodeling genes such as *MMP12* and *MMP25* and downregulation of genes associated with VSMC integrity and metabolic homeostasis, including *SPEG*, *ITGA8*, *KCNA5*, and *SLC25A4*.

Together, these findings support oxidative stress as an amplifier of immune-mediated remodeling in AAA tissue. Leukocyte-derived ROS, mitochondrial redox dysfunction, and innate danger-sensing pathways may strengthen inflammatory signaling and promote downstream proteolytic and VSMC injury, providing a transition from immune activation to structural wall degeneration.

### 3.4. Loss of VSMC and Matrix Homeostasis

In parallel with immune activation and oxidative stress, AAA tissue demonstrated suppression of transcriptional programs involved in VSMC contractility, cell–matrix adhesion, cytoskeletal organization, mitochondrial metabolism, and ECM maintenance. This pattern suggests aneurysmal remodeling is driven by inflammatory injury and loss of the structural and reparative programs required to preserve aortic wall integrity [68,69,70]. In this framework, immune–redox activation converges on VSMC dysfunction and ECM destabilization as key structural features of aneurysmal degeneration.

Several of the most downregulated genes identified in this analysis are closely linked to VSMC homeostasis. *SPEG* contributes to cytoskeletal organization and smooth muscle differentiation, while *ITGA8* mediates cell–matrix adhesion and supports maintenance of the differentiated smooth muscle phenotype [71,72,73,74,75]. Downregulation of *KCNA5* suggests altered membrane potential regulation and contractile responsiveness, whereas reduced *SLC25A4* expression points toward impaired mitochondrial energy exchange and cellular metabolic stability [22,76,77,78]. Collectively, suppression of these genes indicates disruption of coordinated programs required for vascular tone, contractile function, structural organization, and cellular energetics within the aneurysmal wall.

These transcriptional changes are consistent with VSMC phenotypic switching and depletion, both of which are central features of AAA pathology. In response to inflammatory cytokines, oxidative stress, and ECM injury, VSMC may transition away from a contractile, matrix-maintaining phenotype toward a synthetic, inflammatory, or apoptosis-prone state [68,69,70,79]. This shift reduces the ability of the aortic wall to maintain contractile stability, repair damaged matrix, and resist progressive dilation [69,80]. In this context, the observed downregulation of smooth muscle-associated structural and metabolic genes likely reflects both loss of differentiated smooth muscle cell identity and reduced abundance or function of resident VSMC populations.

The ECM findings further reinforce this pattern of structural destabilization. Upregulation of matrix remodeling genes such as *MMP12* and *MMP25* suggests increased proteolytic activity within aneurysmal tissue, while reduced expression of matrix-stabilizing or protease-inhibitory genes such as *FBLN5* and *TIMP4* suggests impaired counter-regulation of ECM degradation. This imbalance favors degradation of elastin and collagen, fragmentation of elastic lamellae, and weakening of the medial layer. Because elastin and collagen provide the tensile strength and recoil necessary for aortic wall stability, disruption of these matrix components provides a direct mechanistic link between the inflammatory transcriptomic signature and progressive aneurysmal dilation [81,82]. 

ECM degradation also may reinforce the inflammatory environment. Proteolytic breakdown of matrix components can generate bioactive fragments that further stimulate macrophage recruitment and inflammatory signaling, while infiltrating leukocytes release additional proteases and ROS that accelerate matrix injury [83]. In this setting, VSMC dysfunction may further limit effective repair by reducing the cellular capacity to maintain and remodel the extracellular scaffold.

Overall, suppression of vascular smooth muscle and matrix maintenance programs provides the structural counterpart to the inflammatory and redox signatures identified above. These findings suggest AAA tissue is characterized by active immune-mediated injury, impaired preservation of the contractile, and ECM architecture required for aortic wall stability.

### 3.5. Upstream Regulatory Convergence: Cytokine and Innate Sensing Networks

Upstream regulator analysis provided an integrative framework for interpreting the coordinated transcriptional programs identified in AAA tissue. Rather than implicating isolated downstream genes, the predicted regulators converged on higher-order inflammatory and innate immune sensing networks, including *IFNG*, *TNF*, *STING1*, *LPS*-like signaling, and poly[I:C]-like signaling. Together, these predictions suggest the aneurysmal transcriptome resembles a state of sustained cytokine activation, PRR engagement, and danger-associated immune signaling.

Predicted activation of *IFNG* and *TNF* supports a central role for cytokine-driven transcriptional remodeling in AAA tissue. Both regulators are capable of coordinating broad inflammatory programs involving leukocyte recruitment, macrophage activation, antigen presentation, adhesion molecule expression, and protease induction [84]. In the context of the present findings, their predicted activation provides a plausible regulatory explanation for the enrichment of leukocyte extravasation, phagocytosis, *TREM1* signaling, Th1/Th2 pathways, and matrix remodeling genes. This suggests cytokine signaling may serve as a central organizing force linking immune cell activation to vascular wall injury.

The predicted activation of *STING1* further implies cytosolic danger-sensing pathways in aneurysmal remodeling. *STING1* links cellular stress, mitochondrial dysfunction, oxidative stress, and cytosolic nucleic acid sensing to downstream interferon and inflammatory cytokine signaling. This is particularly relevant given the redox-associated findings in this study, including upregulation of *NCF1* and *UCP2*, as well as the broader enrichment of innate immune and inflammatory pathways. In this framework, *STING1* may represent a stress-responsive node through which cellular injury amplifies immune activation within the aneurysmal wall.

Predicted activation of *LPS* and poly[I:C] should be interpreted carefully. These upstream regulator predictions do not provide direct evidence of bacterial infection, viral infection, or pathogen exposure in AAA tissue. Instead, they indicate that the observed expression profile resembles transcriptional programs typically induced by microbial pattern recognition and nucleic acid-sensing pathways. This distinction is important because sterile vascular injury can activate overlapping innate immune pathways through damage-associated molecular patterns (DAMP), mitochondrial DNA release, ECM fragments, oxidative stress, and cell death-associated signals [85,86]. Thus, these predictions support the presence of pathogen-sensing-like inflammatory signaling without implying an infectious etiology.

In contrast, several predicted inhibited regulators, including dexamethasone, *CITED2*, *IRGM*, *IRF2BP2*, and *TREX1*, suggest reduced activity of anti-inflammatory, stress-adaptive, autophagy-related, and nucleic acid-regulatory programs. *TREX1* is particularly notable because it normally limits cytosolic DNA accumulation and cGAS-STING activation; its predicted inhibition is directionally consistent with *STING1*-associated inflammatory activation [87]. These inhibited regulators complement the activated cytokine and innate sensing signals by suggesting that inflammatory activation occurs in the setting of impaired counter-regulatory control.

Importantly, upstream regulator analysis is inferential and should not be interpreted as proof that these regulators are causally activated or inhibited in vivo. These predictions are based on the similarity between observed gene expression patterns and curated transcriptional responses associated with known regulators. However, the convergence of cytokine regulators, innate immune sensing signals, and inhibited anti-inflammatory programs is consistent with the differential expression and canonical pathway findings in this study. Together, these results support a regulatory model in which AAA tissue is shaped by sustained cytokine signaling, sterile innate immune activation, and inadequate counter-regulatory restraint.

### 3.6. Impaired Immunoregulatory Restraint and Failed Resolution

In addition to activation of inflammatory and innate immune sensing pathways, AAA tissue demonstrated evidence of impaired immunoregulatory restraint. Canonical pathway analysis showed predicted inhibition of IL-10 signaling and PD-1/PD-L1 checkpoint signaling, while upstream regulator analysis identified strong predicted inhibition of dexamethasone-associated transcriptional activity. Together, these findings suggest the aneurysmal wall is characterized by inflammatory activation and insufficient counter-regulatory signaling to limit or resolve that inflammation. 

The predicted inhibition of IL-10 signaling is particularly relevant because IL-10 is a major anti-inflammatory cytokine that restrains macrophage activation, limits pro-inflammatory cytokine production, and promotes resolution of immune responses. In the context of AAA, reduced IL-10 pathway activity may permit persistence of macrophage- and neutrophil-associated inflammatory programs, including cytokine amplification, phagocytic activation, ROS generation, and matrix-degrading protease expression [88]. This finding complements the observed enrichment of neutrophil degranulation, leukocyte extravasation, phagosome formation, *TREM1* signaling, and PRR signaling, suggesting inflammatory effector pathways are activated in the setting of inadequate anti-inflammatory feedback.

Similarly, predicted inhibition of PD-1/PD-L1 checkpoint signaling suggests reduced immune checkpoint restraint within aneurysmal tissue. PD-1/PD-L1 signaling normally limits excessive T-cell activation and helps maintain peripheral immune tolerance [89]. Its predicted inhibition may therefore contribute to sustained adaptive immune activity, consistent with enrichment of Th1 and Th2 signaling and upregulation of immune-associated genes such as *CCR7* and *CD83*.

Predicted inhibition of dexamethasone-associated transcriptional activity provides an additional systems-level signal of impaired inflammatory suppression [90]. This finding should not be interpreted as evidence of systemic glucocorticoid deficiency or patient medication exposure. Rather, it indicates that the aneurysmal transcriptome is directionally opposed to a glucocorticoid-suppressed inflammatory state, aligning with the broader pattern of activated cytokine signaling, innate immune sensing, and leukocyte effector activity identified throughout the analysis.

Together, these findings support the concept that AAA progression may reflect failed inflammatory resolution as much as excess inflammatory activation. In a normally resolving immune response, anti-inflammatory cytokines, immune checkpoints, efferocytosis, matrix repair programs, and cellular stress response mechanisms help terminate inflammation and restore tissue homeostasis [91,92,93,94,95,96,97]. However, in aneurysmal tissue, predicted inhibition of IL-10 signaling, PD-1/PD-L1 signaling, and dexamethasone-like transcriptional programs suggest that these regulatory mechanisms may be insufficient to counterbalance persistent immune activation.

Overall, these results suggest AAA is not simply an inflammatory disease, but a disease of unresolved inflammation. The combined activation of leukocyte effector pathways and suppression of immunoregulatory signals supports a model in which the aneurysmal wall loses the ability to appropriately terminate immune activation and restore vascular homeostasis. This failure of resolution may help explain how inflammatory remodeling becomes chronic and self-sustaining in AAA.

### 3.7. Integrated Model of AAA Pathogenesis

Collectively, these findings support an integrated model in which AAA pathogenesis reflects a self-reinforcing cycle of immune activation, oxidative stress, ECM degradation, VSMC dysfunction, and impaired inflammatory resolution. Across differential expression, canonical pathway enrichment, and upstream regulator modeling, aneurysmal tissue demonstrated activation of leukocyte recruitment, neutrophil and macrophage effector programs, cytokine signaling, redox-associated injury, and matrix remodeling pathways, accompanied by suppression of vascular contractile programs, ECM organization, and immunoregulatory signaling.

In this model, vascular injury, cellular stress, ECM damage, or danger-associated signals may activate innate immune sensing pathways within the aortic wall [98,99]. Predicted activation of *IFNG*, *TNF*, *STING1*, *LPS*-like signaling, and poly[I:C]-like signaling suggests aneurysmal tissue adopts a transcriptional state resembling sustained cytokine and PRR activation. These upstream signals likely promote recruitment and activation of neutrophils, macrophages, antigen-presenting cells, and lymphocyte populations, consistent with enrichment of leukocyte extravasation, neutrophil degranulation, phagocytosis, *TREM1* signaling, and Th1/Th2 immune pathways.

Once recruited into the aneurysmal wall, inflammatory cells may amplify tissue injury through release of ROS, proteolytic enzymes, cytokines, and chemokines [100]. Upregulation of *NCF1* links phagocyte activation to NOX2-associated oxidative burst, while increased *UCP2* suggests altered mitochondrial redox regulation within the inflammatory microenvironment [15,65]. In parallel, upregulation of matrix remodeling genes such as *MMP12* and *MMP25* connects leukocyte activation to proteolytic degradation of the ECM [101]. These processes may damage elastin and collagen, increase oxidative stress, and generate additional injury-associated signals that reinforce immune activation.

This inflammatory–oxidative environment appears to converge on loss of vascular smooth muscle and ECM homeostasis. Downregulation of genes involved in smooth muscle cell contractility, cell–matrix adhesion, cytoskeletal organization, and mitochondrial energy metabolism, including *SPEG*, *ITGA8*, *KCNA5*, and *SLC25A4*, suggests disruption of the cellular programs required to maintain vascular tone and structural stability [38,75,76,102]. At the same time, reduced expression of matrix-stabilizing or protease-inhibitory genes such as *FBLN5* and *TIMP4* suggests impaired capacity to preserve or repair the extracellular scaffold [103,104]. Together, these changes shift the aortic wall away from homeostatic maintenance and toward progressive structural weakening.

**Figure 3:**
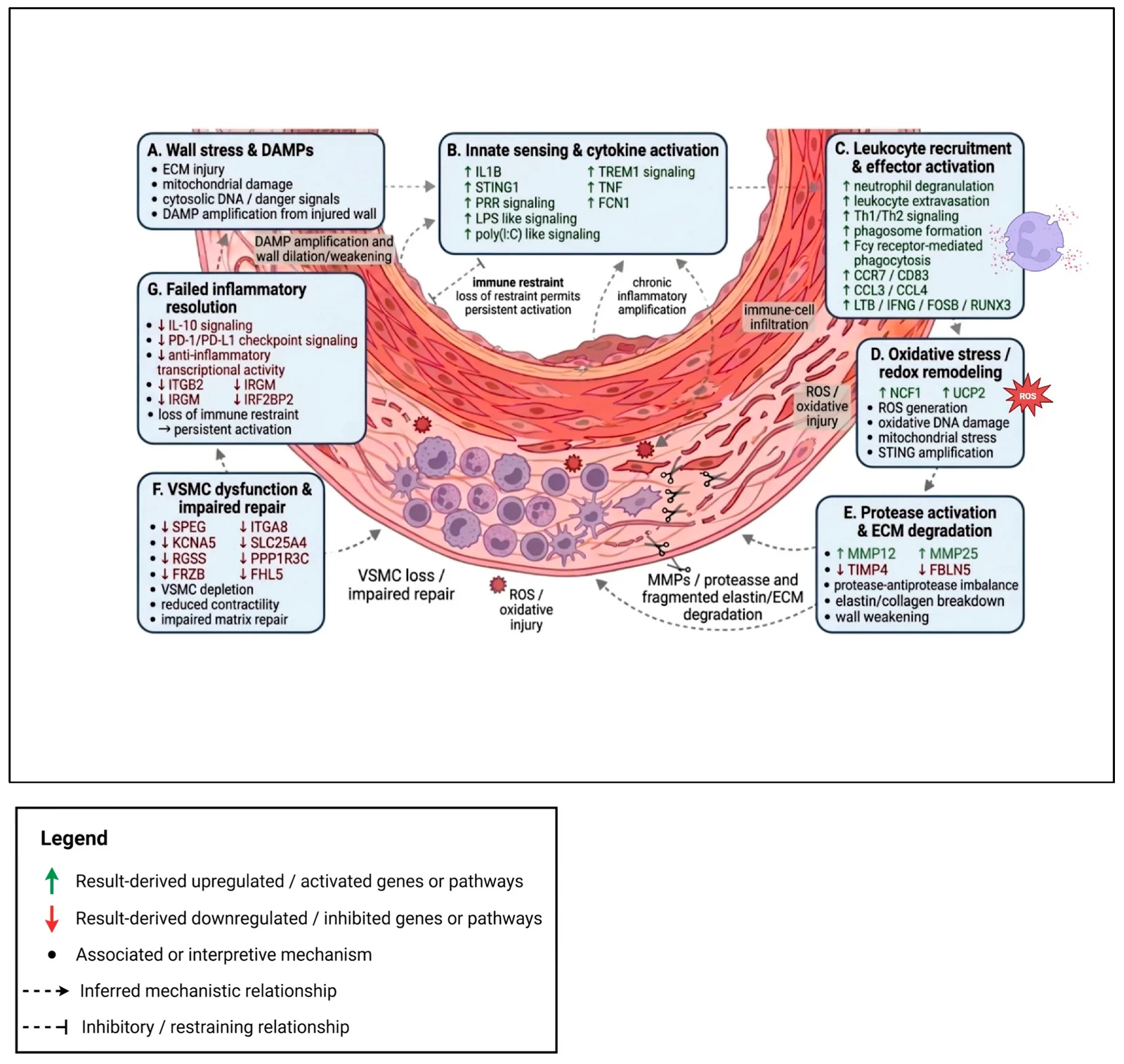
Hypothetical schematic of immune–redox remodeling and impaired vascular wall homeostasis in AAA. This proposed model integrates the differential expression findings from the STARGEO meta-analysis with pathway and network predictions generated through IPA. The schematic illustrates how increased inflammatory signaling, oxidative stress, and ECM, together with suppression of VSMC contractile, matrix maintenance, and immunoregulatory programs, may contribute to progressive vascular wall instability. The illustrated connections represent predicted or literature-supported biological relationships and were not directly tested in this study; therefore, the figure should not be interpreted as an empirically validated causal pathway map. Created in BioRender. Engel, M. (2026) https://BioRender.com/bu57h44 (accessed on 31 July 2026).

A central feature of this model is failed inflammatory resolution. Predicted inhibition of IL-10 signaling, PD-1/PD-L1 checkpoint signaling, and dexamethasone-associated transcriptional activity suggests that counter-regulatory pathways may be insufficient to restrain ongoing immune activation. In the absence of effective immunoregulatory control, leukocyte recruitment, cytokine production, oxidative stress, and protease activity may persist rather than resolve. This creates a feed-forward loop in which inflammation promotes matrix degradation, amplifying immune activation, and, in turn, VSMC dysfunction limits structural repair, as shown in Figure 3 [86,88].

Overall, the present findings support a model of AAA as an immune–redox remodeling disease characterized by persistent inflammatory activation, oxidative amplification, ECM failure, and loss of vascular smooth muscle integrity. This framework emphasizes that disease progression is unlikely to result from a single pathway alone, but rather from convergence of inflammatory, oxidative, proteolytic, structural, and failed resolution programs that collectively destabilize the aortic wall.

Activated immune cells may contribute to oxidative stress and redox remodeling through increased *NCF1* and *UCP2*, promoting ROS generation, oxidative DNA damage, mitochondrial stress, and further STING-related inflammatory amplification. Concurrent upregulation of *MMP12* and *MMP25*, together with downregulation of *TIMP4* and *FBLN5*, supports a protease–antiprotease imbalance that may drive elastin and collagen degradation, ECM fragmentation, and aneurysm wall weakening. Downregulation of VSMC-associated structural, contractile, metabolic, and repair-related genes, including *SPEG*, *ITGA8*, *KCNA5*, *SLC25A4*, *RGS5*, *IGFBP7*, *PPP1R3C*, *FRZB*, and *FHL5*, suggests impaired VSMC function, reduced contractility, and defective matrix repair.

### 3.8. Translational Implications

The transcriptional programs identified in this study have several translational implications for prioritizing biological pathways involved in AAA progression. Importantly, these findings do not identify a single dominant therapeutic target but instead suggest aneurysmal degeneration is driven by coordinated inflammatory–oxidative networks involving cytokine signaling, innate immune sensing, leukocyte effector activity, redox imbalance, protease activation, and impaired structural repair. This pathway-level convergence may help explain why therapies directed at isolated downstream mediators had limited clinical success [105] and suggests future approaches may need to target broader regulatory networks or specific molecular endotypes of disease.

One implication is that inflammatory cytokine and innate immune sensing pathways may represent candidate axes for therapeutic investigation. Upregulation of *IL1B* and predicted activation of *TNF* and *IFNG* suggest cytokine amplification is a central feature of the aneurysmal transcriptome, with potential contributions to leukocyte recruitment, macrophage and neutrophil activation, VSMC dysfunction, and induction of matrix-degrading proteases [47,105,106,107,108]. In parallel, upregulation of *NCF1* and predicted activation of *STING1* highlight redox-inflammatory signaling and cytosolic danger sensing as additional areas for future study [27,98,109]. Together, these findings suggest modulation of cytokine signaling, pathological ROS generation, mitochondrial injury, or sterile innate immune activation may be relevant in biologically selected AAA subsets, although their safety, specificity, and efficacy in human disease require further validation.

Protease-mediated matrix degradation represents another translationally relevant axis. Increased expression of *MMP12* and *MMP25*, with reduced expression of matrix-stabilizing or protease-inhibitory genes such as *FBLN5* and *TIMP4*, suggest a protease–antiprotease imbalance favoring ECM breakdown. However, prior broad MMP inhibition strategies have been limited by lack of specificity and adverse effects [110,111,112]. Future approaches should focus on selective modulation of disease-relevant protease networks, their upstream inflammatory drivers, or restoration of endogenous matrix-stabilizing programs rather than nonspecific suppression of matrix remodeling.

These results also may inform biomarker development by reinforcing several biologically plausible marker classes already under investigation in AAA while nominating additional tissue-derived candidates for validation. Markers of inflammation, oxidative stress, protease activity, and vascular wall homeostasis may provide complementary information to traditional clinical measures such as aneurysm diameter and growth rate, although no circulating biomarker has yet been established for routine AAA diagnosis, surveillance, or rupture prediction [4]. Candidate signals emerging from this analysis include previously studied inflammatory and proteolytic mediators, such as *IL1B* [46], *MMP12*, and related matrix remodeling pathways [113,114], as well as immune trafficking, redox-associated, structural, and metabolic genes including *CCR7*, *CD83*, *FCN1* [44], *NCF1* [27], *UCP2* [115], *SLC25A4* [41], *ITGA8*, *SPEG*, *FBLN5* [103], and *TIMP4* [104]. Because these signals were derived from tissue-level transcriptomics, future studies will be needed to determine whether they correspond to circulating proteins, imaging correlates, spatially localized disease activity, or longitudinal predictors of aneurysm growth and rupture risk [116,117].

A broader translational implication is that AAA may not represent a single molecular disease state. The coexistence of inflammatory, oxidative, proteolytic, and smooth muscle depletion signatures suggests distinct molecular endotypes may exist, with some aneurysms dominated by active immune infiltration, some by redox-mediated injury, and others by advanced structural failure. Defining these endotypes could explain heterogeneity in aneurysm growth and rupture risk and may eventually support more individualized treatment strategies. In this context, transcriptomic profiling may be most valuable when integrated with spatial localization, cell-type-specific analysis, circulating biomarkers, imaging features of wall inflammation, and longitudinal clinical outcomes.

Overall, the translational value of these findings lies in their identification of convergent disease networks rather than isolated gene targets. These implications remain hypothesis-generating, and functional validation will be required before the pathways identified here can be considered clinically actionable.

### 3.9. Limitations

Several limitations should be considered when interpreting these findings. This analysis relied on bulk transcriptomic data, which cannot distinguish whether observed expression differences reflect changes in cellular composition, altered transcriptional states within specific cell populations, or both. This is particularly relevant in AAA tissue, where immune cell infiltration, and VSMC depletion and phenotypic switching are central disease features. As a result, upregulation of immune-associated genes may reflect expansion of infiltrating leukocyte populations, increased activation of those cells, inflammatory reprogramming of resident vascular cells, or a combination of these processes.

The analyzed samples were derived from human aneurysmal tissue, likely representing established or advanced disease; therefore, the identified transcriptional programs may reflect late-stage remodeling rather than the earliest molecular events that initiate aneurysm formation. Although inflammatory activation, oxidative stress, protease activity, and VSMC loss may contribute to progression, cross-sectional tissue analysis cannot determine whether these pathways are initiating drivers, secondary consequences of wall degeneration, or markers of disease severity. Prospective validation studies with expanded sample collection and integration with more granular clinical data describing aneurysm growth over time will be needed to clarify these temporal relationships.

Heterogeneity across datasets may influence the observed transcriptional signals. The included studies were generated using distinct microarray platforms and may have differed in patient characteristics, aneurysm size, medication exposure, smoking history, comorbidities, tissue processing, anatomic sampling, and control tissue source. In particular, AAA specimens were obtained during elective surgical resection, whereas non-aneurysmal control specimens were obtained from brain-dead organ donors or autopsy specimens. Brain death, peri-mortem physiological stress, postmortem interval, and tissue ischemia may independently alter inflammatory and cellular stress response gene expression. Because these factors were not uniformly reported across the included datasets or incorporated into the STARGEO meta-analysis, some of the observed immune activation and stress response signatures may reflect procurement-related differences rather than AAA biology alone. Control aortic tissue may also not perfectly represent healthy infrarenal abdominal aorta, and differences in sampling depth or vessel wall region could affect gene expression patterns. Although meta-analysis across independent human datasets improves robustness and emphasizes reproducible signals, residual heterogeneity and confounding related to tissue procurement remain important limitations.

Differential expression thresholds were based on nominal statistical significance and experimental log ratio cutoffs, which increases susceptibility to false-positive findings at the individual gene level. For this reason, isolated gene-level findings should be interpreted cautiously. The primary strength of the analysis lies in the convergence of related genes, canonical pathways, and upstream regulator predictions rather than in any single differentially expressed transcript. Future studies should validate prioritized genes and pathways in independent cohorts using more stringent statistical approaches and orthogonal methods such as quantitative polymerase chain reaction, immunohistochemistry, spatial transcriptomics, proteomics, or functional assays.

IPA canonical pathway and upstream regulator analyses are inference-based tools that rely on curated biological knowledge and known transcriptional relationships. These methods can identify coordinated pathway patterns and predicted regulatory states, but they do not prove in vivo pathway activation, causal regulation, or direct exposure to specific stimuli. For example, predicted activation of LPS or poly[I:C] should not be interpreted as evidence of bacterial or viral infection; these predictions indicate transcriptional resemblance to microbial pattern or nucleic acid-sensing responses. Similarly, predicted inhibition of dexamethasone-associated activity reflects a gene expression pattern opposed to glucocorticoid-like anti-inflammatory signaling, not necessarily patient glucocorticoid exposure or deficiency.

This analysis focused on transcriptomic data, and it cannot establish whether observed mRNA changes translate into corresponding protein abundance, enzymatic activity, or functional pathway activation. This limitation is particularly important for MMP, cytokines, immune checkpoints, and redox enzymes where post-transcriptional regulation, protein localization, and activity state strongly influence biological function. Integration with proteomics, phosphoproteomics, enzymatic assays, and spatial protein-level validation are necessary to determine the functional relevance of the transcriptional programs identified here.

### 3.10. Future Directions

Future studies should prioritize cell-type-specific and spatial validation of the major inflammatory–oxidative axes identified in this analysis. Single-cell and spatial transcriptomic approaches could determine whether key signals such as *IL1B*, *NCF1*, *MMP12*, *MMP25*, *CCR7*, *CD83*, *UCP2*, and *STING1*-associated programs arise primarily from neutrophils, macrophages, antigen-presenting cells, lymphocytes, VSMC, endothelial cells, adventitial fibroblasts, or localized multicellular niches within the aneurysmal wall. This would help clarify whether the observed bulk tissue signature reflects immune infiltration alone or coordinated communication between inflammatory and resident vascular compartments.

Longitudinal and clinically annotated studies also will be essential. Future analyses should evaluate whether inflammatory–oxidative transcriptional signatures correlate with aneurysm diameter, expansion rate, symptoms, rupture risk, thrombus burden, wall inflammation on imaging, or postoperative outcomes. Such work could determine whether these pathways are markers of advanced disease or clinically meaningful predictors of progression. Integration of tissue transcriptomics with circulating biomarkers, imaging features, and biomechanical modeling may ultimately improve risk stratification beyond aneurysm diameter alone.

Finally, functional validation will be required to determine whether the pathways identified here are therapeutically actionable. Experimental models should test whether modulation of cytokine signaling, NOX2-associated oxidative stress, *STING1*-mediated innate immune sensing, neutrophil effector programs, MMP activity, or pro-resolution immunoregulatory pathways can alter aneurysm initiation, progression, or rupture susceptibility. Attention should be given to interventions that restore balance rather than broadly suppress inflammation, as effective disease modification may require reducing pathologic immune–redox injury while preserving necessary repair and host defense mechanisms.

Together, these future directions may translate the systems-level findings of this meta-analysis into a more precise understanding of AAA biology. By integrating bulk transcriptomics with spatial, single-cell, proteomic, functional, and longitudinal clinical data, future work can refine molecular endotypes, identify clinically relevant biomarkers, and determine which inflammatory–oxidative pathways are true drivers of disease progression.

## 4. Methods

### 4.1. Gene Expression Dataset Identification and Selection

**Figure 4:**
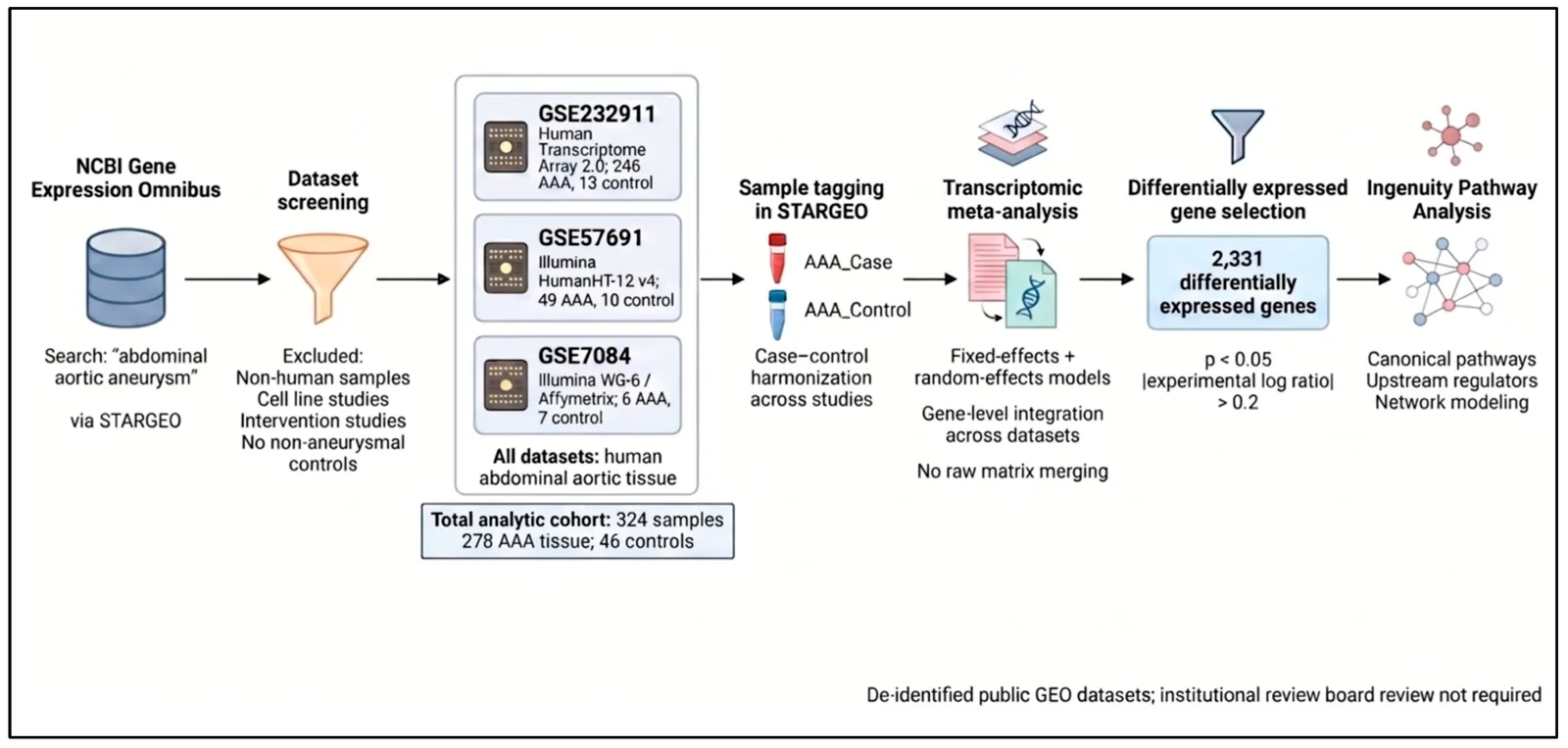
Dataset identification and transcriptomic meta-analysis methods. Created in BioRender. Engel, M. (2026) https://www.biorender.com/ (accessed on 31 July 2026).

Gene expression data were sourced from the NCBI’s GEO, a publicly available repository of high-throughput genomic datasets. A free-text search for “abdominal aortic aneurysm” was performed using STARGEO, a web-based platform designed for integrative transcriptomic meta-analysis. Methodology is summarized in Figure 4.

Datasets were excluded if they contained non-human samples, cell line-based data, samples exposed to experimental interventions or pharmacological treatments that could alter baseline gene expression, or single-class datasets lacking appropriate non-aneurysmal control samples. Following exclusion criteria, three independent human AAA transcriptomic datasets generated from distinct microarray platforms remained: GSE232911 [118], GSE57691 [119], and GSE7084 [120].

### 4.2. Study Cohort and Sample Characteristics

The final analytic dataset comprised 324 total samples, including 278 AAA tissue samples and 46 non-aneurysmal control aortic tissue samples. Aneurysmal tissue samples were obtained from patients undergoing elective surgical repair of AAA, whereas control samples were derived from non-AAA. Within STARGEO, aneurysmal samples were tagged as “AAA_Case” and non-aneurysmal samples as “AAA_Control” to enable case–control comparisons across studies. Additional dataset and sample-level details are summarized in Table 2.

### 4.3. Transcriptomic Meta-Analysis

Transcriptomic meta-analysis was performed using STARGEO, which integrates gene-level differential expression results across independent GEO datasets using fixed-effects and random-effects statistical models [121]. Integration occurs at the gene level, enabling cross-platform analysis without merging raw expression matrices. Meta-analysis *p*-values generated by STARGEO were used to identify differentially expressed genes.

Genes demonstrating statistical significance (*p* < 0.05) and an absolute experimental log ratio > 0.2 were retained for downstream analyses. Because *p*-values were not adjusted for multiple testing, integration across independent cohorts and subsequent pathway and network analyses were used to prioritize concordant biological programs rather than isolated single gene effects. Using these criteria, a total of 2331 differentially expressed genes were identified and included in subsequent pathway and network analyses.

To assess the sensitivity of the gene-level findings to multiple testing correction, the Benjamini–Hochberg procedure was retrospectively applied to the meta-analysis *p*-values for all 22,112 genes evaluated. Genes with an FDR-adjusted *p*-value ≤ 0.05 and an absolute experimental log ratio > 0.2 were considered significant in the sensitivity analysis. The original 2331 gene set was retained for the primary IPA analyses, whereas the FDR-adjusted results were used to evaluate the stability of prioritized individual gene findings. FDR-adjusted *p*-values are provided in Table S1, and the complete list of all 22,112 differentially expressed genes with FDR-adjusted *p*-values is provided in Table S4.

### 4.4. Pathway, Upstream Regulator, and Network Analyses

Functional and pathway analyses were performed using IPA (Qiagen, Hilden, Germany), a curated bioinformatics platform that contextualizes gene expression data using experimentally validated findings from scientific literature and publicly available biological datasets. Differentially expressed genes were analyzed using canonical pathway enrichment and upstream regulator analysis.

### 4.5. Ethical Considerations

All analyses were conducted on de-identified, publicly available data from the NCBI GEO and were exempt from the University of Central Florida and Nemours Children’s Health Institutional Review Board review.

## 5. Conclusions

This transcriptomic meta-analysis of human AAA tissue identifies a reproducible inflammatory–oxidative transcriptional program characterized by immune activation, redox-associated signaling, protease-mediated ECM remodeling, and suppression of VSMC contractile and structural gene expression. Convergent evidence from differential expression, canonical pathway enrichment, and upstream regulator modeling supports a systems-level framework in which cytokine amplification, innate immune sensing, oxidative stress, impaired immunoregulatory restraint, and loss of vascular wall homeostasis collectively shape the aneurysmal microenvironment.

Together, these findings support a model of AAA as an immune–redox remodeling disease in which inflammatory, oxidative, proteolytic, structural, and failed resolution programs converge to destabilize the aortic wall. Although these results remain hypothesis-generating and require functional validation, they provide a mechanistic foundation for prioritizing pathway-directed therapeutic strategies, biomarker development, and molecular endotyping in AAA. Future integration of single-cell, spatial, proteomic, functional, and longitudinal clinical data will be necessary to define the cellular sources, temporal dynamics, and clinical relevance of these transcriptional programs and to translate these molecular insights into actionable strategies for risk stratification and disease modification.

## Figures and Tables

**Table 1:** Top 10 differentially expressed genes in AAA tissue identified by STARGEO. Positive values indicate upregulation in AAA tissue, whereas negative values indicate downregulation relative to controls.

Upregulated Genes	Experimental Log Ratio	FDR-Adjusted *p*-Value	Downregulated Genes	Experimental Log Ratio	FDR-Adjusted *p*-Value
*FOSB* (FosB proto-oncogene, AP-1 transcription factor subunit)	1.538	0.128695	*PPP1R3C* (protein phosphatase 1 regulatory subunit 3C)	−1.609	0.008702
*NCF1* (neutrophil cytosolic factor 1)	1.337	0.100799	*SPEG* (striated muscle enriched protein kinase)	−1.367	0.042164
*IL1B* (interleukin 1 beta)	1.239	0.078053	*TCEAL2* (transcription elongation factor A-like 2)	−1.367	0.132249
*FCN1* (ficolin 1)	1.215	0.132970	*SCRG1* (stimulator of chondrogenesis 1)	−1.275	0.125032
*CD83* (cluster of differentiation 83)	1.129	0.074711	*RASL12* (RAS-like family 12)	−1.247	0.087221
*MMP25* (matrix metallopeptidase 25)	1.089	0.183400	*SLC25A4* (solute carrier family 25 member 4)	−1.245	0.110967
*UCP2* (uncoupling protein 2)	1.058	0.003574	*ITGA8* (integrin subunit alpha 8)	−1.241	0.007595
*LTB* (lymphotoxin beta)	1.057	0.046067	*FRZB* (frizzled-related protein)	−1.238	0.062514
*MMP12* (matrix metallopeptidase 12)	1.020	0.016735	*FHL5* (four and a half LIM Domains 5)	−1.184	0.004936
*CCR7* (C-C motif chemokine receptor 7)	1.012	0.065338	*KCNA5* (potassium voltage-gated channel subfamily A member 5)	−1.181	0.017550

**Table 2:** Characteristics of gene expression series included in the AAA transcriptomic meta-analysis.

GEO Series	Platform	Tissue Type	Samples Included in Meta-Analysis	Tissue Source	Notes/Reference
GSE232911	Human Transcriptome Array 2.0 (Thermo Fisher Scientific)	Abdominal aortic wall (intimal–medial and adventitial layers), sampled from the anterior vessel wall in intraluminal thrombus-covered and, when available, intraluminal thrombus-free regions.	AAA: 246 aneurysmal abdominal aortic wall tissue samples derived from 76 patients (intraluminal thrombus-covered regions in all patients; intraluminal thrombus-free regions in 34 patients). Controls: 13 non-aneurysmal abdominal aortic wall tissue samples.	AAA: Elective open abdominal aortic aneurysm repair. Controls: Abdominal aorta from beating-heart, brain-dead transplant donors.	Multiple tissue samples per patient; intraluminal thrombus-covered wall designated as primary aneurysmal tissue [118].
GSE57691	Illumina HumanHT-12 v4 Expression Beadchip	Full-thickness abdominal aortic wall tissue.	AAA: 49 aneurysmal abdominal aortic wall tissue samples, including 20 small AAA (mean AAA diameter 53.4 ± 2.3 mm) and 29 large AAA (mean AAA diameter 68.4 ± 14.3 mm). Controls: 10 non-aneurysmal abdominal aortic wall tissue samples.	AAA: Open surgical repair for AAA. Controls: Abdominal aorta from beating-heart, brain-dead transplant donors.	Samples include full-thickness abdominal aortic wall tissue from small and large abdominal aortic aneurysms profiled using bulk tissue microarrays [119].
GSE7084	Illumina Sentrix Human WG-6 and Affymetrix whole-genome expression arrays	Full-thickness abdominal aortic wall tissue.	AAA: 6 aneurysmal abdominal aortic wall tissue samples. Controls: 7 non-aneurysmal abdominal aortic wall tissue samples.	AAA: Abdominal aortic aneurysm repair surgery. Controls: Autopsy-derived non-aneurysmal abdominal aorta collected within 24 h of death.	Gene expression data were generated using two microarray platforms. Analysis was restricted to genes represented on both platforms to enable cross-platform integration [120].

## Data Availability

The data analyzed in this study are available in the Gene Expression Omnibus (GEO) at https://www.ncbi.nlm.nih.gov/geo/ (accessed on 25 October 2024) repository under accession numbers GSE232911, GSE57691, and GSE7084.

## Supplementary Materials

The following supporting information can be downloaded at https://www.mdpi.com/article/10.3390/ijms27167257/s1.

## Abbreviations

AAA	Abdominal aortic aneurysm
ECM	Extracellular matrix
MMP	Matrix metalloproteinase
VSMC	Vascular smooth muscle cells
NCBI	National Center for Biotechnology Information
GEO	Gene Expression Omnibus
STARGEO	Search, Tag, Analyze Resource for GEO
IPA	Ingenuity Pathway Analysis
*FOSB*	FosB proto-oncogene, ap-1 transcription factor subunit
*NCF1*	Neutrophil cytosolic factor 1
*IL1B*	Interleukin 1 beta
*FCN1*	Ficolin 1
*CD83*	Cluster of differentiation 83
*MMP25*	Matrix metallopeptidase 25
*UCP2*	Uncoupling protein 2
*LTB*	Lymphotoxin beta
*MMP12*	Matrix metallopeptidase 12
*CCR7*	C-C motif chemokine receptor 7
*PPP1R3C*	Protein phosphatase 1 regulatory subunit 3C
*SPEG*	Striated muscle enriched protein kinase
*TCEAL2*	Transcription elongation factor A-like 2
*SCRG1*	Stimulator of chondrogenesis 1
*RASL12*	RAS-like family 12
*SLC25A4*	Solute carrier family 25 member 4
*ITGA8*	Integrin subunit alpha 8
*FRZB*	Frizzled-related protein
*FHL5*	Four and a half LIM domains 5
*KCNA5*	Potassium voltage-gated channel subfamily A member 5
ROS	Reactive oxygen species
NADPH	Nicotinamide adenine dinucleotide phosphate
NET	Neutrophil extracellular trap
*RUNX3*	Runt-related transcription factor 3
RGS5	Regulator of G-protein signaling 5
IGFBP2	Insulin-like growth factor binding protein 2
FBLN5	Fibulin-5
TIMP4	Tissue inhibitor of metallopeptidase inhibitor 4
PRR	Pattern recognition receptor
*IFNG*	Interferon-γ
*TNF*	Tumor necrosis factor
*STING1*	Stimulator of interferon response cGAMP interactor 1
*CITED2*	Cbp/p300 interacting transactivator with Glu/Asp rich carboxy-terminal domain 2
*IRGM*	Immunity-related GTPase M
*IRF2BP2*	Interferon regulatory factor 2 binding protein 2
*TREX1*	Three prime repair exonuclease 1
DAMP	Damage-associated molecular patterns
